# Preliminary investigation of potential links between pigmentation variants and opioid analgesic effectiveness in horses during cerebrospinal fluid centesis

**DOI:** 10.1186/s12917-024-04139-z

**Published:** 2024-07-12

**Authors:** Elouise K. Bacon, Callum G. Donnelly, Rebecca R. Bellone, Bianca Haase, Carrie J. Finno, Brandon D. Velie

**Affiliations:** 1 Equine Genetics and Genomics Group, School of Life and Environmental Sciences, University of Sydney, Sydney, NSW Australia; 2 Department of Population Health and Reproduction, School of Veterinary Medicine, University of California, Davis, CA 95616 USA; 3 Department of Clinical Sciences, College of Veterinary Medicine, Cornell University, Ithica, NY 14850 USA; 4 Veterinary Genetics Laboratory, School of Veterinary Medicine, University of California, Davis, CA 95616 USA; 5 School of Veterinary Science, University of Sydney, Sydney, NSW Australia

**Keywords:** Horse, Opioid, Sensitivity, Pigmentation, MC1R, ASIP

## Abstract

**Background:**

The pleiotropic effects of the melanocortin system show promise in overcoming limitations associated with large variations in opioid analgesic effectiveness observed in equine practice. Of particular interest is variation in the *melanocortin-1-receptor* (*MC1R*) gene, which dictates pigment type expression through its epistatic interaction with the *agouti signalling protein* (*ASIP*) gene. *MC1R* has previously been implicated in opioid efficacy in other species; however, this relationship is yet to be explored in horses. In this study, analgesic effectiveness was scored (1-3) based on noted response to dura penetration during the performance of cerebrospinal fluid centisis after sedation and tested for association with known genetic regions responsible for pigmentation variation in horses.

**Results:**

The chestnut phenotype was statistically significant (*P* < 0.05) in lowering analgesic effectiveness when compared to the bay base coat colour. The 11bp indel in ASIP known to cause the black base coat colour was not significant (*P*>0.05); however, six single nucleotide polymorphisms (SNPs) within the genomic region encoding the ASIP gene and one within MC1R were identified as being nominally significant (*P*<0.05) in association with opioid analgesic effectiveness. This included the location of the known e MC1R variant resulting in the chestnut coat colour.

**Conclusions:**

The current study provides promising evidence for important links between pigmentation genes and opioid effectiveness in horses. The application of an easily identifiable phenotype indicating variable sensitivity presents a promising opportunity for accessible precision medicine in the use of analgesics and warrants further investigation.

**Supplementary Information:**

The online version contains supplementary material available at 10.1186/s12917-024-04139-z.

## Background

To date, clinical studies of equine opioid analgesia have revealed significant variations in effectiveness based on the individual horse and level of pain [1–4]. Both published and anecdotal reports have also highlighted adverse gastrointestinal and central nervous system (CNS) excitatory effects associated with opioid administration, worsened by higher dosage rates [5–12]. While investigations have begun exploring polymorphisms in the Cytochrome 2D6 (*CYP2D6)* gene involved in the metabolism of many opioids in horses, additional studies are required to determine their functional implications for opioid analgesia sensitivity [13, 14]. Moreover, CYP2D6 polymorphisms studied to date do not result in any known easily identifiable phenotypes which, coupled with the high cost associated with pharmacogenetics, limits clinical application.

Early exploration into the pleiotropic effects of the melanocortin system shows promise in overcoming these limitations, with genes involved in melanogenesis shown to impact several additional biological systems, including those involved in pigment expression [15–17]. There are five melanocortin receptor subtypes belonging to the superfamily of G protein-coupled receptors [18]. Of particular interest are the *melanocortin-1-receptor* (*MC1R*) and *agouti signalling protein (ASIP)* genes which are involved in pigment switching. Normally eumelanin is produced when signalling through MC1R is stimulated by the agonist melanocyte stimulating hormone (MSH), belonging to a family of melanocortin peptides which are proteolytically cleaved from the precursor proopiomelanocortin (POMC) via enzyme activity [19]. Phaeomelanin is produced when MC1R signalling is blocked by the antagonist agouti signalling protein (ASIP). A recessive loss of function mutation in the antagonist *ASIP* (11 bp deletion on exon 2), results in eumelanin production only. Red pigment only (pheomelanin) results from one of two known recessive epistatic loss of function missense mutation in *MC1R* (c.248C > T, p.S83F or c.250 > A, p.D84N) [20–22]. Importantly, *MC1R* transcripts and proteins are also expressed in the immune system and central nervous system in the periaqueductal gray matter (PAG) of the midbrain [23, 24]. Both systems have been shown to contain opioid receptors, with the PAG also known to be involved in pain modulation [25–27]. Similar associations have been explored in humans, with subjects expressing *MC1R* variants, known to cause red hair and pale skin, recording an altered pain perception and a greater analgesic response to opioids [28, 29]. However, it is unknown if this association is a direct result of the *MC1R* gene or through other variations within the melanocortin system such as the melanocortin-4-receptor (*MC4R)* gene. The *MC4R* gene, whilst relatively unexplored in horses, has demonstrated extensive pleiotropic effects in rodent models, including energy expenditure, pain processing, and behavioural attributes [18, 30–35]. Transcripts of *MC4R*, its antagonist agouti-related protein (*AGRP*), and the *POMC* gene have all been detected in the dorsal root ganglion and spinal cord, with upregulation in neuropathic rats indicating the involvement of the *MC4R* gene in nociception [36]. Further, rodent models have supported the implication of the *MC4R* neuropeptide in both biochemical and behavioural effects of opioids [37, 38]. As such, this study aimed to investigate if links between opioid analgesic effectiveness and the genetic loci involved in pigmentation (*MCIR*, *ASIP*, *MC4R*, *AGRP*, and *POMC*) are also present in horses. If found to exist, such phenotypic links with opioid analgesic effectiveness could bring the veterinary industry a step closer to precision medicine in equine sedation and pain management.

## Materials and methods

### Phenotype data

Cerebrospinal fluid (CSF) centesis was performed on a subset (*n* = 49) of the Pioneer 100 Horses from the University of California Davis research herd as part of an ongoing project (UC Davis IACUC 21343 & 21,700) [39]. The number of horses used in this study was dictated by the requirements of the primary investigation. The cohort comprised of 27 males and 22 females, made up of warmblood (*n* = 9), thoroughbred (*n* = 13), Quarter horse (*n* = 23), Iberian (*n* = 2), Standardbred (*n* = 1), and Arabian (*n* = 1) breeds. Horse age ranged between 5–20 (median 13), with an average weight of 550 kg (range 400-703 kg). The horses used in this study come from diverse backgrounds and have been donated for research purposes, spending much of their time in paddocks. Clinical nociceptive response data were opportunistically collected during the performance of CSF centesis, noting the pain response of individual horses after sedation. Despite being opportunistic in nature, the collection of this data with the intention of investigating analgesic effectiveness was a critical part of the process and methodology used. Pain responses recorded included, but were not limited to, head-shaking, jumping, and twitching (Table 1). Analgesic effectiveness was then scored on a scale of one to three based on these noted responses (Table 1). To ensure consistency, two examiners noted the behavioural response of the horse in conjunction, neither of which were aware of the use of results for the investigation of coat colour links. Base coat colour phenotypes were determined by visual inspection and photographic record on two occasions (fall and spring) by an expert in phenotyping. This study included 29 bay and 18 chestnut horses, with grey horses (2) excluded from base coat colour association analyses as their *MC1R* and *ASIP* genotypes cannot be identified phenotypically.

**Table 1:** Score corresponding to response description used to score analgesic effectiveness

Score	Description
1	Large reaction, shaking head, jumped, difficulty obtaining sample
2	Mild movement when dura penetrated, twitch/jerk
3	No reaction

Other known or suspected factors that could influence a horse’s response to analgesics were also recorded. This included age, weight, breed, sex, and administered doses of detomidine hydrochloride, xylazine hydrochloride, and hydromorphone hydrochloride. A starting dosage range of 0.01 mg/kg body weight intravenously was used in the administration of the opioid hydromorphone, the primary drug of interest in this study. CSF centesis, whereby pain response was recorded upon dura penetration, was performed approximately 8–10 min after the first detomidine administration and 2–3 min following the second, consistent across the cohort. Hydromorphone was administered 5 min after the first detomidine at the second dosage interval 2–3 min before CSF centesis, with additional hydromorphone given to two horses who were highly reactive after first dose. This was factored into analysis, with total hydromorphone administered being used as a covariate. Additional information from the CSF centesis, including collector, date, number of attempts, needle depth, success of the collection, volume, and appearance, was also compiled.

### Genotype data

Blood samples were collected from all horses. These samples underwent whole-genome sequencing (WGS), with alignment to EquCab3.0 and variants called according to the methodology outlined in Donnelly et al. [40, 41]. WGS data for all horses included in this study are available through the NCBI SRA database (PRJNA841639).

Due to the well documented dictation of pigmentation production by the *ASIP* and *MC1R* genes, their coding sequences were extracted from variant call files (VCFs) using custom scripts in BCFtools [42]. Genomic regions were identified using the National Library of Medicine Genome Data viewer within the EquCab3.0 genome assembly and corresponding literature (*MC1R*: Chr3:g.36979312–36,980,266, *ASIP*: Chr22:g.26009341–26072655) [20, 41]. All horses within the study had previously been genotyped for red factor and agouti mutations, which was used for base coat colour genotype analysis. Based on their suspected interactions with pigmentation expression and reported links to opioid metabolism in other species, the regions containing *MC4R* (Chr8:g.80658134–80659867), *AGRP* (Chr3:g.18612129–18,617,417), and *POMC* (Chr15:g.71778801–71784802) were also extracted for analysis [17]. Loci within the chosen regions were filtered for fixation using custom scripts in the statistical software R, and only variants that were not fixed in the population were kept for future analysis [43].

### Statistical analyses

#### Phenotype analyses

Summary statistics for each coat colour were calculated using basic R scripts. Chi-squared tests of association were performed considering each horse to determine if significant associations exist between the coat colour phenotype and analgesic effectiveness. Base coat colour phenotypes were comparted to genotypes to determine phenotyping accuracy.

#### SNP association analyses

Single nucleotide polymorphisms (SNPs) within each gene, stratified by chromosome, were then tested for association with analgesic effectiveness using a generalized linear model in the SNPassoc package in R. These analyses allow for the addition of covariates and models according to 5 different inheritance patterns (co-dominant, dominant, recessive, over dominant, and additive) [44]. Based on prior literature, breed, sex, age, and dosage rate of hydromorphone were included as covariates in all association analyses, with analgesic effectiveness as the dependent variable^1−3,8,18,46^. Nominal (*p* < 0.05) and Bonferroni significance levels were used to determine significance. Generalized linear models were then fit and D-Squared values were calculated to determine the variance explained by each significant SNP at each locus, both before and after the addition of covariates.

#### Haplotype analyses

Haplotype analyses were performed for all SNPs identified to have at least nominally significant (*P* > 0.05) association with analgesic effectiveness. Association between haplotype and analgesic effectiveness was tested by a generalized linear model using the haplo.stats package in R [46]. The models also included the effects of significant covariates from the previous analyses.

## Results

**Fig. 1:**
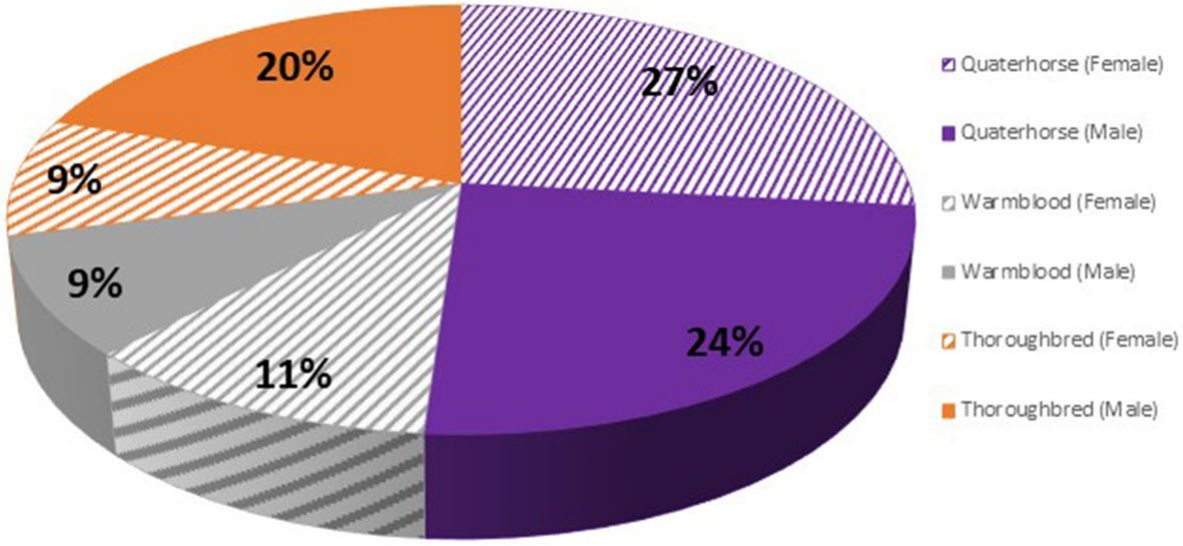
Distribution of sex, stratified by breed^†^. Purple = Quarter horse (*n* = 23), Grey = Warmblood (*n* = 9), Orange = Thoroughbred (*n* = 13), Stripes = female, Solid = male^†^. Iberian, Standardbred, & Arabian horses were excluded (*n* = 4)

**Fig. 2:**
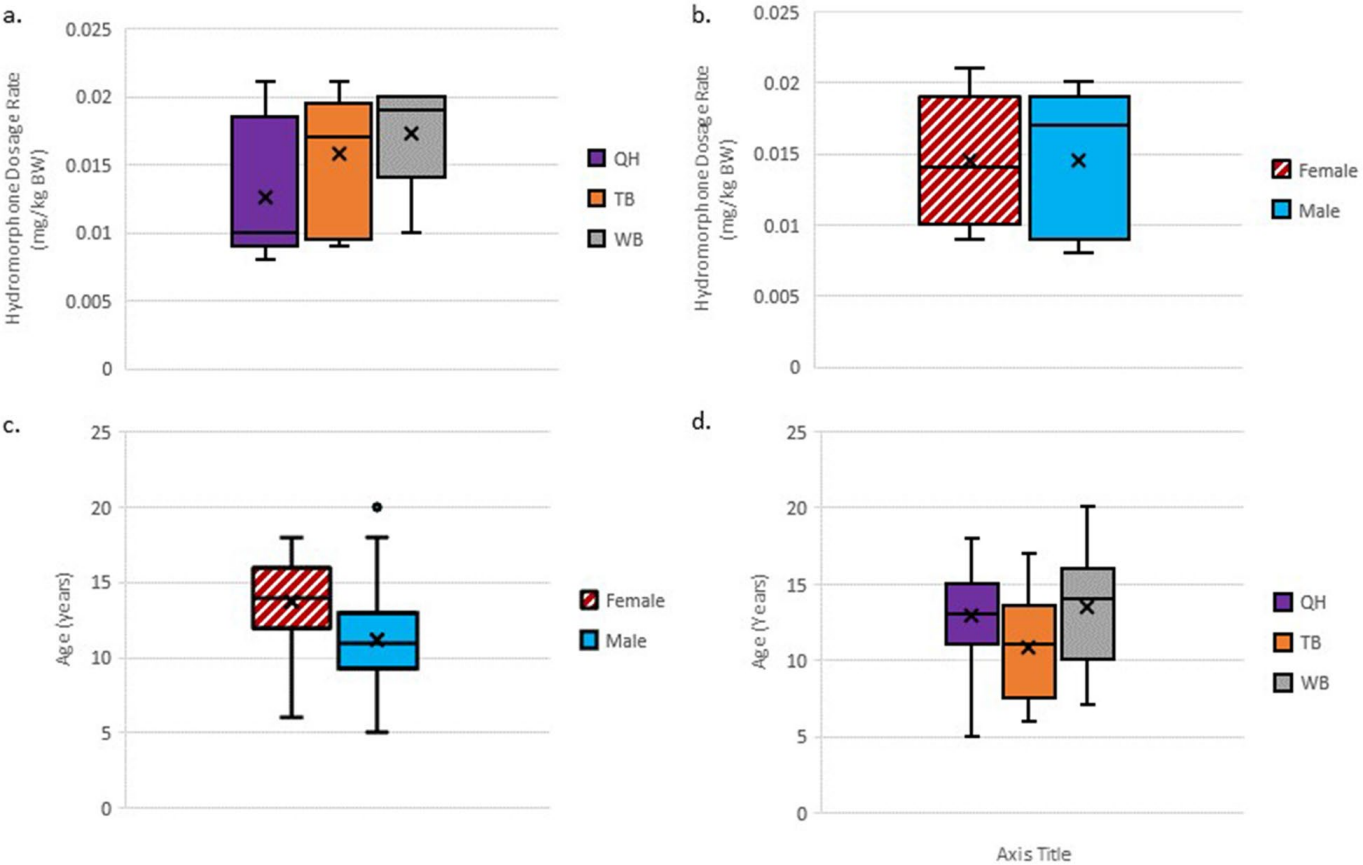
Boxplots showing a) Hydromorphone dosage rate^†^ stratified by breed^‡^, b) Hydromorphone dosage rate^†^ stratified by sex, c) age (in years) stratified by sex, and d) age (in years) stratified by analgesic effectiveness score. Purple = Quarter horse, Grey = Warmblood, Orange = Thoroughbred, Stripes = female, Solid = male., Black = analgesic effectiveness score 1, Pink = analgesic effectiveness score 2, Brown = analgesic effectiveness score 3.^†^ Target range 0.01–0.02 mg/kg BW intravenously. ‡ Iberian, Standardbred, & Arabian horses were excluded (*n* = 4)

**Fig. 3:**
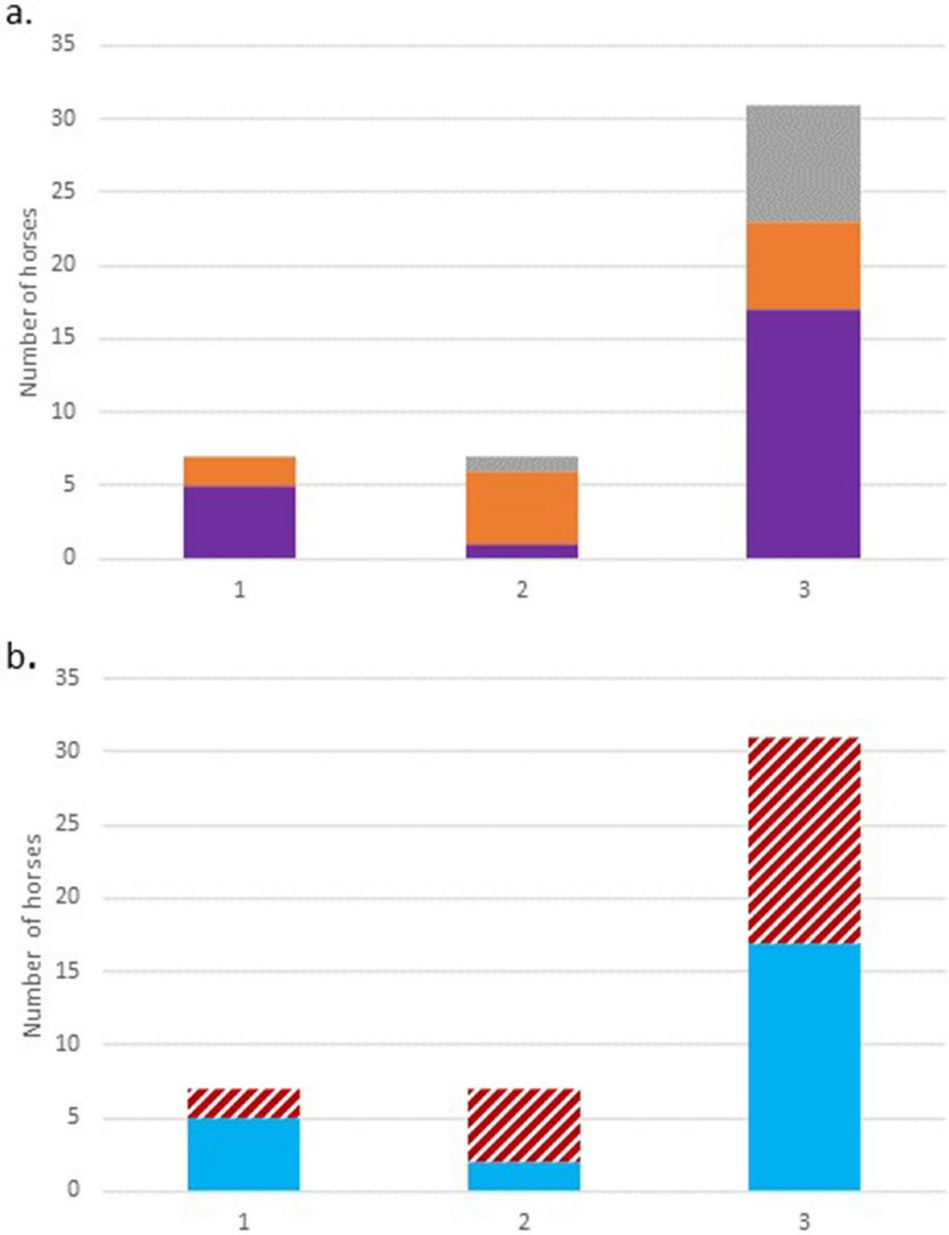
Distribution of analgesic effectiveness (AE) stratified by breed^†^ (a) and sex (b). ^†^ Iberian, Standardbred, & Arabian horses were excluded (*n* = 4). Grey = Warmblood, Orange = Thoroughbred, Purple = Quarter horse, Stripes (red) = female, Solid (blue) = male

Across the sampled population, six breeds were represented, with males and females comprising 53% and 47% of the samples, respectively (Fig. 1). The age range of horses included in the study was 5–20 years old with an average of 12, and the average dosage rate of hydromorphone was 0.014 mg/kg of body weight (Fig. 2). The mean recorded analgesic effectiveness was 2.49 (Fig. 3). Observed base coat colour phenotypes showed 100% correlation with corresponding genotypes. Two greys were present in the cohort which were excluded from base coat colour analyses. When chestnut horses were looked at in isolation, males recorded a lower average analgesic effectiveness score; however, the sample size was too small to draw reliable conclusions (Supplementary 1).

When evaluated independently, neither *MC1R* or *ASIP* genotypes were statistically significant in relation to analgesic effectiveness (*P* > 0.05); however the homozygous dominant E/E *MC1R* genotype recorded a greater mean and median analgesic effectiveness score, trending to more as the presence of the recessive e allele increases (Tables 2 and 3). The other known *MC1R* mutation (c.250G > A, p.D84N) giving the recessive e^a^ allele was not present within the sampled cohort [47]. The base coat colour determined by phenotype observations revealed chestnuts to have a significantly (*P* < 0.05) lower analgesic effectiveness score (Table 4).

**Table 2:** Analgesic effectiveness and dosage rates stratified by *MC1R* genotype observed in this cohort. Chi-squared *p*-value = 0.08. Analgesic effectiveness scored one (least effective) to three (most effective)

*MC1R Genotypes*	*Mean analgesic effectiveness*	*Median analgesic effectiveness*	*Mean dosage rate (mg/kg BW)*	*Median dosage rate (mg/kg BW)*
E/E (*n* = 11)	2.8	3	0.015	0.017
E/e (*n* = 20)	2.7	3	0.015	0.016
e/e (*n* = 18)	2.1	2	0.014	0.01

**Table 3:** Analgesic effectiveness and dosage rates stratified by *ASIP* genotype. Chi-squared P-value = 0.07. Analgesic effectiveness scored one (least effective) to three (most effective)

*ASIP Genotypes*	*Mean analgesic effectiveness*	*Median analgesic effectiveness*	*Mean dosage rate (mg/kg BW)*	*Median dosage rate (mg/kg BW)*
A/A (*n*=23)	2.4	3	0.014	0.012
A/a (*n*=23)	2.6	3	0.015	0.017
a/a (*n*=3)	2.3	2	0.017	0.02

**Table 4:** Analgesic effectiveness and dosage rates stratified by base coat colour. Chi-squared *p*-value = 0.023

*Coat colour phenotype*	*Mean analgesic effectiveness*	*Median analgesic effectiveness*	*Mean dosage rate (mg/kg BW)*	*Median dosage rate (mg/kg BW)*
Bay (*n* = 29)	2.7	3	0.014	0.015
Chestnut (*n* = 18)	2.1	2	0.02	0.02

**Fig. 4:**
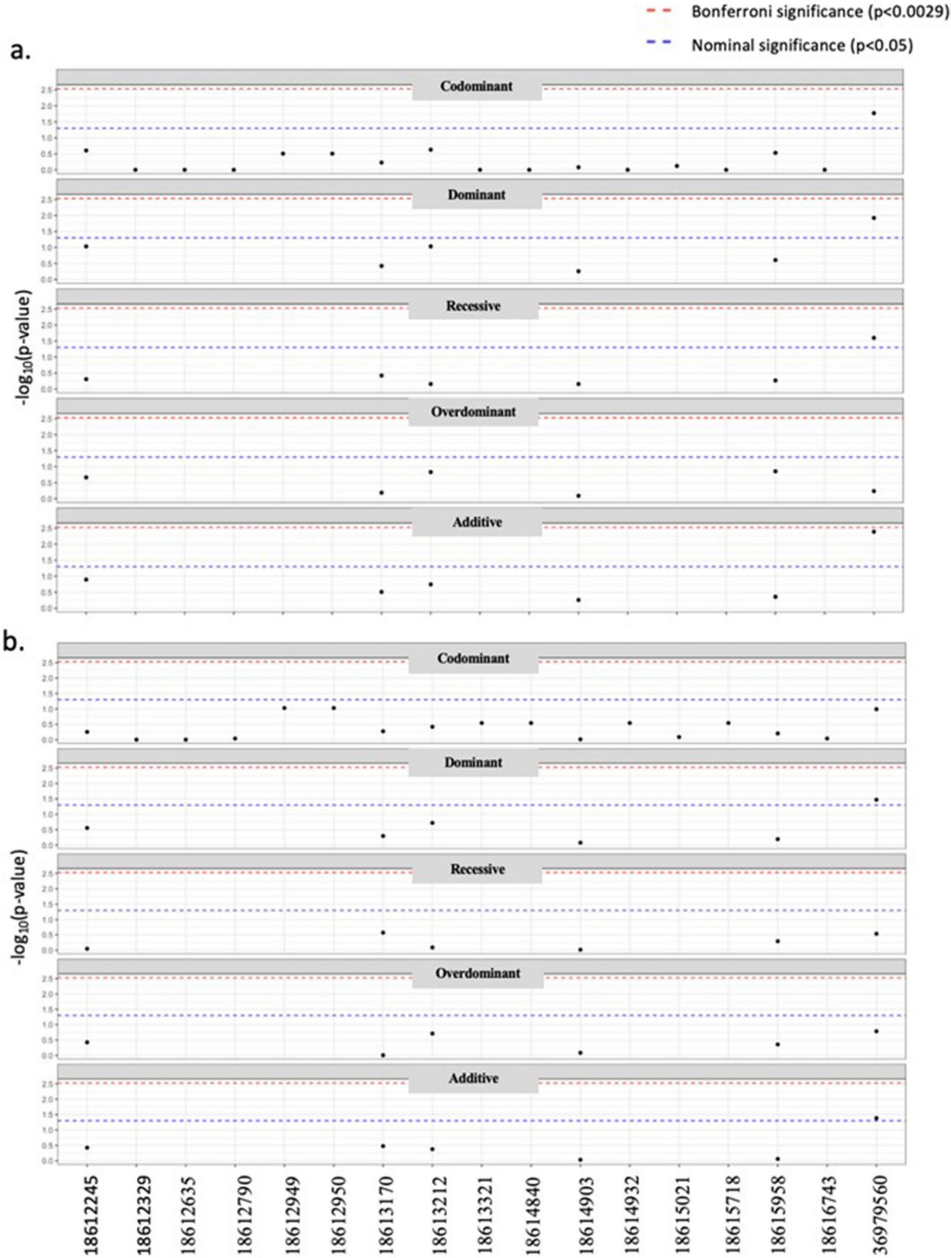
SNP association analysis on chromosome 3 before (a) and after (b) the addition of covariates (age, breed^†^, sex, and hydromorphone dosage rate) using a generalized linear model. The significant *MC1R* location on chromosome 3 (g.36979560) (outlined in red) alone accounted for 14.8% of explained variance before the addition of covariates. Covariates alone accounted for 8.7% of explained variance. The *MC1R* SNP with the addition of covariates accounted for 18%. ^†^ Iberian, Standardbred, & Arabian horses were excluded (*n* = 4)

**Fig. 5:**
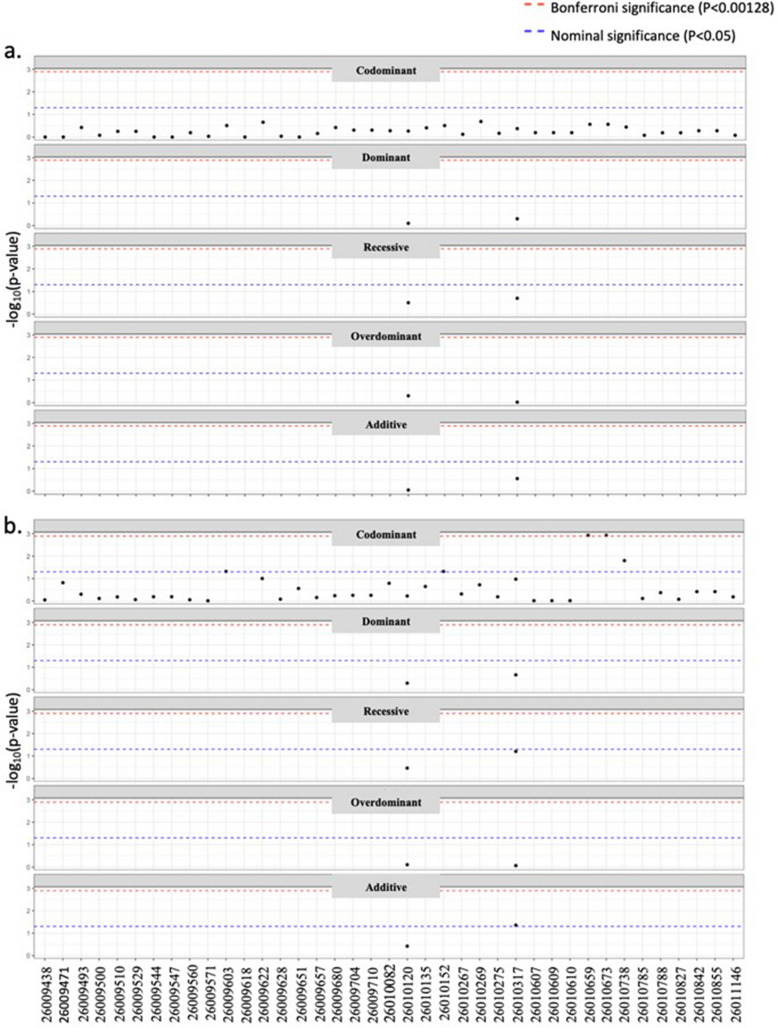
SNP association analysis on chromosome 22 before (a) and after (b) the addition of covariates (age, breed^†^, sex, and hydromorphone dosage rate) using generalizes linear model. *ASIP* significant SNPs alone accounted for 15% of variation before the addition of covariates. *ASIP* significant SNPs in addition to covariates accounted for 45% explained variance. ^†^ Iberian, Standardbred, & Arabian horses were excluded (*n* = 4)

After filtering out fixed locations (i.e. locations with only a single variant), 123 locations remained for evaluation (Supplementary 2). Lack of representation from Arabian, Standardbred, and Iberian breeds (≤ 2 horses each) resulted in these breeds (*n* = 4 horses) being excluded from the analyses. Association analyses with analgesic effectiveness across all 123 SNPs, stratified by chromosome, identified six nominally significant SNPs within *ASIP*, and one within *MC1R* (Fig. 4; Fig. 5; Supplementary 3; Supplementary 4). The established location of the 11 bp deletion (NC_009165.3) resulting in the recessive a allele was not represented by any of the SNPs identified to be significant within the *ASIP* genomic region. Two of the six SNPs within the *ASIP* gene passed Bonferroni threshold for significance after the addition of covariates (age, sex, breed and hydromorphone dosage rate) (Fig. 5). One SNP within the *MC1R* gene was nominally significant; however, it did not pass the Bonferroni threshold (Fig. 4). D-squared values assigned 15% and 14.8% of explained variance to *ASIP* and *MC1R* significant SNPs, respectively, before the addition of covariates (Fig. 4; Fig. 5). Significant *ASIP* SNPs in conjunction with covariates were responsible for 45% of variation, compared to 18% attributed to the *MC1R* SNP with covariates added.

The six SNPs included in the haplotype analyses for the *ASIP* gene are listed in Table 5. Significant differences in analgesic effectiveness were present between haplotypes both before and after the addition of covariates (Fig. 5). The *MC1R* significant location (Chr3:g.36979560) was also included as a covariate in the *ASIP* haplotype analysis due to their known epistatic interaction. The common haplotype (TTATCC) was significant in this model, as with the dominant CC *MC1R* genotype (Table 6).

**Table 5:** Significant SNPs identified from association analyses

*Chromosome *	*Gene*	*Location *	*P-value *	*Model of significance *	*Variant *	*Consequence *
22	*ASIP*	26009603	0.048	Codominant	rs3434477505	Upstream gene variant
22	*ASIP*	26010152	0.048	Codominant	rs1143791359	Intron variant
22	*ASIP*	26010317	0.044	Log-additive	rs782880240	Intron variant
22	*ASIP*	26010659	0.001	Codominant	rs3432049684	5 prime UTR variant
22	*ASIP*	26010673	0.001	Codominant	rs3436135903	5 prime UTR variant
22	*ASIP*	26010738	0.016	Codominant	rs3431324242	Missense variant
3	*MC1R*	36979560	0.034	Dominant	rs68458866	Missense variant

**Table 6:** Haplotype analysis of significant SNPs within the Agouti signalling protein gene

*Haplotype*^a^	*Frequency*	*diff*	*P-value *^Ƹ^
*Original model*			
TTGTCC	0.489	2.26	-
TTATCC	0.267	0.34	0.040*
TTGATA	0.200	0.42	0.035*
Rare	0.045	-0.80	0.035*
*MC1R*^b ^*covariate model*			
TTGTCC	0.491	1.93	
TTATCC	0.265	0.39	0.016*
TTGATA	0.198	0.32	0.141
	0.046	- 0.51	0.208
* MC1R*C/T		0.39	0.095
* MC1R*C/C		0.76	0.008*
*All covariate*^c ^*included model*			
TTGTCC	0.500	1.79	-
TTATCC	0.256	0.58	0.013*
TTGATA	0.198	1.08	0.004**
Rare	0.046	0.02	0.962
* MC1R* C/T	-	0.33	0.139
* MC1R *T/T	-	0.16	0.620
DR Hyd^d^	-	-22.02	0.316
Male	-	-0.67	0.061
Thoroughbred	-	0.16	0.641
Warmblood	-	0.87	0.047*
Age	-	0.02	0.491

^Ƹ^Generalised linear model

*Passing nominal significance threshold (*P*<0.05)

**Passing Bonferroni significance threshold (*P*<0.0045

^a^Included locations in order of inclusion: Chr22:g.26009603, Chr22:g.26010152, Chr22:g.26010317, Chr22:g.26010659, Chr22:g.26010673, & Chr22:g.26010738.

^b^Inclusion of Chr3; g.36979560 as covariate indicating the *MC1R* mutation

^c^Covariate included model with *MC1R* genotype, age, breed, sex and hydromorphone dosage rate

^d^Dosage rate of Hydromorphone (mg/kg BW)

## Discussion

In this study, the potential association of pigmentation genes with opioid analgesic effectiveness in the horse was explored, with a primary focus on the *MC1R, MC4R, ASIP, AGRP*, and *POMC* genetic regions. Analyses revealed significant associations between SNPs within the *ASIP* and *MC1R* genes, and analgesic effectiveness.

Results from this study suggest that both genes responsible for the type of melanin production may also be involved in opioid response. However, this effect is thought to be melanin independent, with literature indicating altered opioid sensitivities to be through interactions with both the transmission of pain and expression with opioid receptors [48]. Previous literature has identified *MC1R* immunoreactivity and mRNA in conjunction with opioid receptors within both the periaqueductal grey matter pain modulating descending pathway and in some immune cells in other species [17]. Association analyses of SNPs within the MC1R genomic region identified one SNP of significance on chromosome 3 at g.36979560, which is the location of the known missense mutation in *MC1R* (c.248C > T, p.S83F), that results in the recessive e allele [24, 49]. Covariates had a relatively small effect of explained variation when added to the model, accounting for a 3.2% increase. Analysis of the *MC1R* genotype alone saw mean analgesic effectiveness decrease further with each recessive e allele (Table 2). However, it is important to note no *MC1R* genotype was statistically significant (*P* > 0.05) in chi-squared testing, potentially owing to the opportunistic nature and small sample size of this study. Despite this, there was a noticeably lower mean and median analgesic effectiveness score of the e/e genotype as compared to E/E, supported by the significantly (*P* < 0.05) lower analgesic effectiveness in chestnuts compared to bay horses revealed in base coat colour analysis (Table 4). In looking at both the genotype and phenotype analyses, these results suggest a possible additive effect of the recessive e MC1R allele, with sample size potentially limiting the statistical significance.

Interestingly, the results of this study indicate an inverse opioid-*MC1R* relationship in horses to those seen in other species. Human and rodent studies have demonstrated *MC1R* loss of function variants exhibit increased opioid analgesia and varied pain tolerance opposite to that seen in this study [29, 50]. Yet when looking at MC1R involvement in pain alone in dogs a greater nociceptive sensitivity to mechanical force was recorded in dogs with a single variant [51]. Notably, no evidence has found an association between the MC1R variant and coat colour in dogs. Nevertheless, this evidence suggests some species variation which may play a role in the varied results of this study. Previous studies in other species have also indicated sex to play a large role in opioid interaction, with the *MC1R* gene shown to only mediate kappa-opioid analgesia in female mice [45]. However, when looking at analgesic responsiveness to the mu-opioid receptor agonist similar to hydromorphone in MC1R non-functional mice and humans, sex did not appear to have any effect [29]. Whilst results here indicate some interaction of sex in the chestnut population sampled, no conclusion can be drawn due to the small sample size. The identified significance of sex in similar *MC1R* studies in other species may explain the loss of significance after the addition of covariates. Additionally, the *MC1R* genotype has previously been implicated in pain modulation and sensitivity, with varying results for different nociceptive modalities [28, 29]. These results indicate the possibility that altered analgesic effectiveness may be attributed to pain sensitivity, either independently or in conjunction with opioid interactions.

The contrasting result of this study from those in other species may also be attributed to the interactions of genetic variations with the non-opioid analgesics administered in conjunction with the opioid hydromorphone. As a consistent plane of sedation was achieved in all horses to safely facilitate CSF centesis, it can be inferred that differences seen in response to noxious stimuli were in part a result of different analgesic response to hydromorphone. However, it is still important to consider the potential contributions of the other drugs administered as non-opioids and sedatives are also known to increase pain thresholds in some animals. When comparing the minimum alveolar concentrations of anaesthetic that prevented movement in response to noxious stimuli, *MC1R* mutant mice had on average a 5.5% increase across 4 different inhalation anaesthetics [49]. Similarly, observations of red-haired women saw them require significantly more desflurane, an inhalation anaesthetic, and lidocaine, a synthetic local anaesthetic, than dark-haired women [28, 52]. Whilst none of the administered analgesics in this study were inhalant anaesthetics, this evidence indicates the *MC1R* gene to be involved in the modulation of analgesia and anaesthetics beyond opioids. Subsequently, compounding interactions between varying pain sensitivity and multiple analgesic administrations resulting from the opportunistic nature of this investigation potentially skewed the outcome of measurable opioid efficacy. Nevertheless, despite the small sample size seen in this investigation, the significant association with *MC1R* genotype and base coat colour (chestnut) along with the association in *MC1R* genotype and altered analgesic effectiveness highlights pigmentation expression as a potential phenotypic marker for opioid sensitivity that warrants further investigation. If confirmed to exist, the application of the chestnut phenotype associated with the *MC1R* genetic variant could help in improving the precision of equine pain management and reduce the incidence of side effects, having substantial safety implications for both veterinarians and horses.

In analyses of the *ASIP* genomic region, six SNPs within the *ASIP* genetic region were statistically significant. This significance was seen after the addition of covariates to the model indicating positive confounding, potentially with breed. All SNPs identified are intergenic variants from the European Variation Archive release (5); however, no functional effects have been documented to date. Furthermore, when looking at haplotype analysis of these variants with the *MC1R* mutation as the sole covariate, only the common haplotype (TTATCC) is significant, whilst the interaction of the dominant CC *MC1R* genotype was also significant (Table 6). Whilst this is somewhat unsurprising given the epistatic interactions between *ASIP* and *MC1R*, it is important to note that the SNPs and associated haplotypes do not include the known location of the 11 bp deletion that results in the recessive “a” *ASIP* allele. When looked at in isolation, there was no significant association between the causative *ASIP* allele and analgesic effectiveness (Table 3). The SNPs and haplotypes identified as significant may be resultant from signatures of genetic factors beyond the region sequenced, or unannotated aspects of the region, rather than the individual SNPs, warranting further association studies. Nevertheless, the significance of unexplored SNPs within the *ASIP* genomic region highlights an area for further investigation to better understand the relationship between pigmentation variants and opioid analgesia.

Whilst little published literature exists on the interaction of opioids with the *ASIP* gene, in horses nor other species, increased diversity in pigmentation expression resulting from domestication may have inadvertently selected for altered pain tolerance. The bay coat colour (dictated by the dominant ASIP allele) is thought to be the earliest pigmentation expressed by horses prior to domestication and the introduction of analgesic intervention by humans [53–55]. Later coat colours, including black (resultant from an 11pb deletion in exon 2 within *ASIP*) and chestnut (*MC1R;* c.248C > T, p.S83F, p.D84N), increased in prevalence due to artificial selection resultant from domestication. In a rodent study, Dark-Agouti rats (RT1^av1^) have been shown to require higher doses of buprenorphine, a partial mu-opioid agonist, over Sprague–Dawley and August Copenhagen Irish rats [56, 57].

The significance of *ASIP* SNPs*,* seen both in isolation and in conjunction with the *MC1R* gene, could also be due to the antagonistic relationship of ASIP with MC1R. Analyses of base coat colour saw the bay phenotype yielding significantly higher analgesic effectiveness scores than the chestnut phenotype (Table 4). *ASIP* protein binds to melanocortin-1-receptors in conjunction with *AGRP*, preventing G-protein release and subsequently the production of cyclic adenosine monophosphate (cAMP) [58]. Activation of opioid receptors similarly results in the inhibition of cAMP production, impeding neurotransmitter release. This inhibition, in conjunction with the ion channel regulatory effects of opioids, results in neural excitability and inhibitory effects of opioid analgesics [48]. The significant SNPs identified within the *ASIP* genomic region, whilst not known to be involved in coat colour expression, may be involved in such processes inhibiting cAMP production, thus effecting opioid sensitivity. Whilst no SNPs within the *AGRP* region were significantly associated, this may have been impacted by the sample size and limited genetic diversity. As such, the interactions of *ASIP*, *MC1R,* and *AGRP* impacting opioid sensitivity requires further investigation in order to gain a deeper understanding.

*MC1R* loss of function variation results in decreased systemic melanocyte-stimulating hormone (MSH) and melanocytic *POMC* transcription. This alters the physiologic antagonism between the central opioid tone mediating receptor OPRM1 and its opposing MSH responsive receptor MC4R [59]. The expression of MC4R, MC1R, and OPRM1 receptors has been identified within the PAG area of the brain, known to be involved in inhibiting ascending nociceptive transmission and eliciting analgesia [60]. However, analysis of MC4R, as well as the POMC and AGRP genetic regions showed no statistical significance (supplementary 3; supplementary 4), thus were not presented within the results. The results seen may be owing to the constraints placed on sample size by the opportunistic nature of this investigation limiting the strength of analyses and subsequently impeding the identification of any genotype effect within these genetic regions.

Subjective scoring of analgesic effectiveness through veterinarian notes taken during CSF centesis further limits the results of this study, as no precise measurements of plasma concentration levels of administered drugs were available. The administration of hydromorphone can induce central nervous system excitation and locomotor behaviour [9]. This result is dependent on dosage rate in horses, hence limiting the ability to accurately assess analgesic effectiveness [8, 9, 61, 62]. Other opioids have also demonstrated intra- and inter-individual variability of anti-nociceptive plasma concentrations in horses higher than that of other species [63]. These combined effects mean further studies into pigmentation expression variants associated with analgesic effectiveness of opioids should include plasma concentrations, nociceptive threshold testing, as well as observations of locomotor activity. Further studies should also consider the duration of sedation as a factor, potentially looking across different modalities of adverse stimulus. *MC1R* variants have demonstrated variable pain thresholds between thermal and electrical stimuli, with the duration of hydromorphone effectiveness also showing stimulus-dependent variations [8, 28, 29].

## Conclusion

The current study provides a basis for the association of genes involved in pigmentation expression and opioid effectiveness in horses, with both the gene responsible for coat colour, *MC1R* and *ASIP,* indicating significant associations with opioid analgesic effectiveness. In both genotypic and phenotypic analyses, chestnut coat colour was associated with lower analgesic efficacy, opposite to that seen in other species. Given the complex nature of opioid analgesic interactions and the limitations placed on this study by its opportunistic nature, further research is required before clinical applications can be seen. However, both the *MC1R* and *ASIP* regions show promise in representing equine opioid analgesic effectiveness, with easily identifiable phenotypes presenting an opportunity to overcome costs typically associated with precision medicine, warranting more targeted and extensive further investigation.

### Supplementary Information

Supplementary Material 1.

## Data Availability

The WGS datasets supporting the conclusions of this article are available in thee NCBI SRA database repository (PRJNA841639).

## Abbreviations

CNS: Central nervous system
CYP2D6: Cytochrome 2D6
POMC: Proopiomelanocortin
MC1R: Melanocortin-1-receptor
ASIP: Agouti signalling protein
MSH: Melanocyte stimulating hormone
PAG: Periaqueductal gray matter
MC4R: Melanocortin-4-receptor
AGRP: Agouti-related protein
CSF: Cerebrospinal fluid
WGS: Whole genome sequencing
VCF: Variant call file
SNP: Single nucleotide polymorphism
cAMP: Cyclic adenosine monophosphate
