# Supplementary material for: Preliminary investigation of potential links between pigmentation variants and opioid analgesic effectiveness in horses during cerebrospinal fluid centesis
Source: BMC Vet Res. 2024 Jul 12;20:311. doi: 10.1186/s12917-024-04139-z (PMC11245827; doi:10.1186/s12917-024-04139-z)
Supplement: Supplementary file 1 — Supplementary Material 1. [file 12917_2024_4139_MOESM1_ESM.docx]

**Supplementary**

Supplementary 1: Analgesic effectiveness score stratified by sex for chestnut horses only. Female mean =2.4, median = 3. Male mean =1.8, median =1

Supplementary 2: SNPs remaining after filtering (n = 123).

^†^ locations based on EquCab3.

| *Gene* | *Chromosome* | *Location*^‡^ | *Consequence* | *Variant* |
| --- | --- | --- | --- | --- |
| *ASIP* | 22 | 26009438 | Upstream gene variant | rs3439886844 |
| *ASIP* | 22 | 26009471 | Upstream gene variant | rs3439964938 |
| *ASIP* | 22 | 26009493 | Upstream gene variant | rs3439677105 |
| *ASIP* | 22 | 26009500 | Unknown variant | NA |
| *ASIP* | 22 | 26009510 | Upstream gene variant | rs3439807470 |
| *ASIP* | 22 | 26009529 | Upstream gene variant | rs3439886937 |
| *ASIP* | 22 | 26009544 | Unknown variant | NA |
| *ASIP* | 22 | 26009547 | Unknown variant | NA |
| *ASIP* | 22 | 26009560 | Upstream gene variant | rs3444677489 |
| *ASIP* | 22 | 26009571 | Upstream gene variant | rs3444872381 |
| *ASIP* | 22 | 26009603 | Upstream gene variant | rs3434477505 |
| *ASIP* | 22 | 26009618 | Unknown variant | NA |
| *ASIP* | 22 | 26009622 | Upstream gene variant | rs3444441720 |
| *ASIP* | 22 | 26009628 | Upstream gene variant | rs3436727260 |
| *ASIP* | 22 | 26009651 | Upstream gene variant | rs3440010191 |
| *ASIP* | 22 | 26009657 | 5 prime UTR variant | rs3434599649 |
| *ASIP* | 22 | 26009680 | 5 prime UTR variant | rs3434922563 |
| *ASIP* | 22 | 26009704 | 5 prime UTR variant | rs3432424138 |
| *ASIP* | 22 | 26009710 | 5 prime UTR variant | rs3432543641 |
| *ASIP* | 22 | 26009793 | 5 prime UTR variant | rs3436027491 |
| *ASIP* | 22 | 26009796 | 5 prime UTR variant | rs3434058204 |
| *ASIP* | 22 | 26009800 | 5 prime UTR variant | rs3436898092 |
| *ASIP* | 22 | 26009816 | 5 prime UTR variant | rs3433717936 |
| *ASIP* | 22 | 26009823 | 5 prime UTR variant | rs3432949546 |
| *ASIP* | 22 | 26009848 | Intron variant | rs3429687909 |
| *ASIP* | 22 | 26009854 | Intron variant | rs3434289924 |
| *ASIP* | 22 | 26009940 | Intron variant | rs3432308330 |
| *ASIP* | 22 | 26009942 | Intron variant | rs3434840476 |
| *ASIP* | 22 | 26009949 | Intron variant | rs3432282746 |
| *ASIP* | 22 | 26009958 | Intron variant | rs396035478 |
| *ASIP* | 22 | 26009975 | Intron variant | rs3432787536 |
| *ASIP* | 22 | 26009986 | Intron variant | rs3437257749 |
| *ASIP* | 22 | 26010008 | Intron variant | rs395363183 |
| *ASIP* | 22 | 26010009 | Intron variant | rs3429661668 |
| *ASIP* | 22 | 26010041 | Intron variant | rs3436305982 |
| *ASIP* | 22 | 26010053 | Intron variant | rs3432413929 |
| *ASIP* | 22 | 26010082 | Intron variant | rs3430980031 |
| *ASIP* | 22 | 26010090 | Intron variant | rs3434477472 |
| *ASIP* | 22 | 26010093 | Intron variant | rs3434504422 |
| *ASIP* | 22 | 26010095 | Intron variant | rs3432424147 |
| *ASIP* | 22 | 26010111 | Intron variant | rs3433005381 |
| *ASIP* | 22 | 26010120 | Intron variant | rs1142882694 |
| *ASIP* | 22 | 26010135 | Intron variant | rs3429863429 |
| *ASIP* | 22 | 26010152 | Intron variant | rs1143791359 |
| *ASIP* | 22 | 26010160 | Intron variant | rs3433723733 |
| *ASIP* | 22 | 26010196 | Intron variant | rs3429648763 |
| *ASIP* | 22 | 26010212 | Intron variant | rs3432870540 |
| *ASIP* | 22 | 26010267 | Unknown variant | NA |
| *ASIP* | 22 | 26010269 | Intron variant | rs3444872563 |
| *ASIP* | 22 | 26010275 | Intron variant | rs3444871506 |
| *ASIP* | 22 | 26010317 | Intron variant | rs782880240 |
| *ASIP* | 22 | 26010607 | 5 prime UTR variant | rs3435411937 |
| *ASIP* | 22 | 26010609 | 5 prime UTR variant | rs3433564742 |
| *ASIP* | 22 | 26010610 | 5 prime UTR variant | rs3429863389 |
| *ASIP* | 22 | 26010659 | 5 prime UTR variant | rs3432049684 |
| *ASIP* | 22 | 26010673 | 5 prime UTR variant | rs3436135903 |
| *ASIP* | 22 | 26010738 | Missense variant | rs3431324242 |
| *ASIP* | 22 | 26010785 | Unknown variant | NA |
| *ASIP* | 22 | 26010788 | Unknown variant | NA |
| *ASIP* | 22 | 26010827 | Missense variant | rs3434508726 |
| *ASIP* | 22 | 26010842 | Missense variant | rs3436865485 |
| *ASIP* | 22 | 26010855 | Missense variant | rs3430829454 |
| *ASIP* | 22 | 26010934 | Synonymous variant | rs1141302083 |
| *ASIP* | 22 | 26010962 | Missense variant | rs1138663336 |
| *ASIP* | 22 | 26010981 | Missense variant | rs1140301480 |
| *ASIP* | 22 | 26011146 | Missense variant | rs1140088841 |
| *ASIP* | 22 | 26011228 | Missense variant | rs1141208805 |
| *ASIP* | 22 | 26011230 | Missense variant | rs1152026978 |
| *ASIP* | 22 | 26011262 | Missense variant | rs1136226730 |
| *ASIP* | 22 | 26011265 | Missense variant | rs1141685802 |
| ASIP | 22 | 26011315 | Synonymous variant | rs1150555157 |
| *AGRP* | 3 | 18612245 | Intron variant | rs396871065 |
| *AGRP* | 3 | 18612329 | 3 prime UTR variant | rs1142413739 |
| *AGRP* | 3 | 18612635 | Intron variant | rs3430682393 |
| *AGRP* | 3 | 18612790 | Synonymous variant | rs3440741491 |
| *AGRP* | 3 | 18612949 | Unknown variant | NA |
| *AGRP* | 3 | 18612950 | Unknown variant | NA |
| *AGRP* | 3 | 18613170 | Intron variant | rs68595092 |
| *AGRP* | 3 | 18613212 | Intron variant | rs395232749 |
| *AGRP* | 3 | 18613321 | 5 prime UTR variant | rs3431275049 |
| *AGRP* | 3 | 18613728 | 5 prime UTR variant | rs1150646354 |
| *AGRP* | 3 | 18614840 | Intron variant | rs3431654325 |
| *AGRP* | 3 | 18614866 | Intron variant | rs3440533131 |
| *AGRP* | 3 | 18614903 | Intron variant | rs394514553 |
| *AGRP* | 3 | 18614932 | Intron variant | rs1145932602 |
| *AGRP* | 3 | 18615021 | Intron variant | rs1150848701 |
| *AGRP* | 3 | 18615247 | Intron variant | rs3444122429 |
| *AGRP* | 3 | 18615377 | Unknown variant | NA |
| *AGRP* | 3 | 18615414 | Intron variant | rs1147259028 |
| *AGRP* | 3 | 18615715 | Unknown variant | NA |
| *AGRP* | 3 | 18615718 | Unknown variant | NA |
| *AGRP* | 3 | 18615797 | Intron variant | rs3443972350 |
| *AGRP* | 3 | 18615833 | Intron variant | rs3439940855 |
| *AGRP* | 3 | 18615958 | Intron variant | rs1144385086 |
| *AGRP* | 3 | 18616473 | Unknown variant | NA |
| *AGRP* | 3 | 18616743 | Intron variant | rs3440722369 |
| *MC1R* | 3 | 36979319 | Missense variant | rs3440125198 |
| *MC1R* | 3 | 36979560 | Missense variant | rs68458866 |
| *MC4R* | 8 | 80658876 | Synonymous variant | rs1146612074 |
| *MC4R* | 8 | 80659100 | Missense variant | rs1138796464 |
| *MC4R* | 8 | 80659347 | Synonymous variant | rs395990674 |
| *MC4R* | 8 | 80659510 | Upstream gene variant | rs1145398955 |
| *POMC* | 15 | 71778960 | 5 prime UTR variant | rs68974374 |
| *POMC* | 15 | 71778981 | 5 prime UTR variant | rs1139072941 |
| *POMC* | 15 | 71779326 | Intron variant | rs394351065 |
| *POMC* | 15 | 71779435 | Intron variant | rs395037318 |
| *POMC* | 15 | 71779532 | Intron variant | rs397035751 |
| *POMC* | 15 | 71779838 | Intron variant | rs1140011756 |
| *POMC* | 15 | 71779885 | Intron variant | rs394321794 |
| *POMC* | 15 | 71780037 | Intron variant | rs1145377593 |
| *POMC* | 15 | 71780643 | Intron variant | rs1135895882 |
| *POMC* | 15 | 71780751 | Intron variant | rs1148639007 |
| *POMC* | 15 | 71781235 | Intron variant | rs1145417140 |
| *POMC* | 15 | 71781416 | Intron variant | rs3099703327 |
| *POMC* | 15 | 71781607 | Intron variant | rs3099703327 |
| *POMC* | 15 | 71781761 | Intron variant | rs3105017673 |
| *POMC* | 15 | 71782321 | Missense variant | rs1145864079 |
| *POMC* | 15 | 71783223 | Unknown variant | NA |
| *POMC* | 15 | 71783549 | Intron variant | rs395784885 |
| *POMC* | 15 | 71783910 | Intron variant | rs3442272747 |
| *POMC* | 15 | 71783911 | Intron variant | rs3442402359 |
| *POMC* | 15 | 71784054 | Synonymous variant | rs1136949726 |
| *POMC* | 15 | 71784555 | Synonymous variant | rs3435807756 |

Supplementary 3: Chromosome analyses of significant SNPs on chromosome 8 before (a) and after (b) the addition of covariates (age, breed^†^, sex, and hydromorphone dosage rate) using generalizes linear model. ^†^ Iberian, Standardbred, & Arabian horses were excluded (n=4)
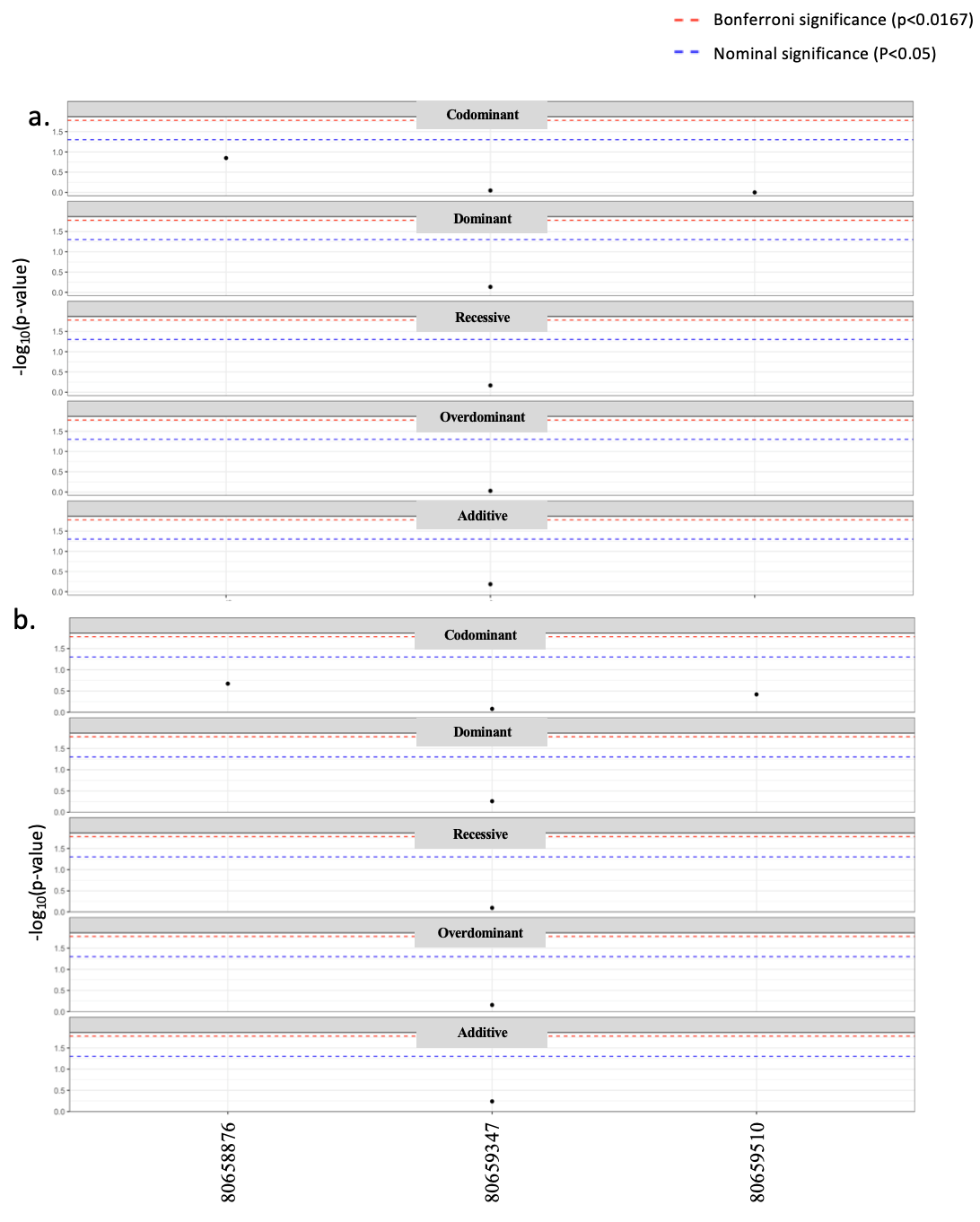


SNP location


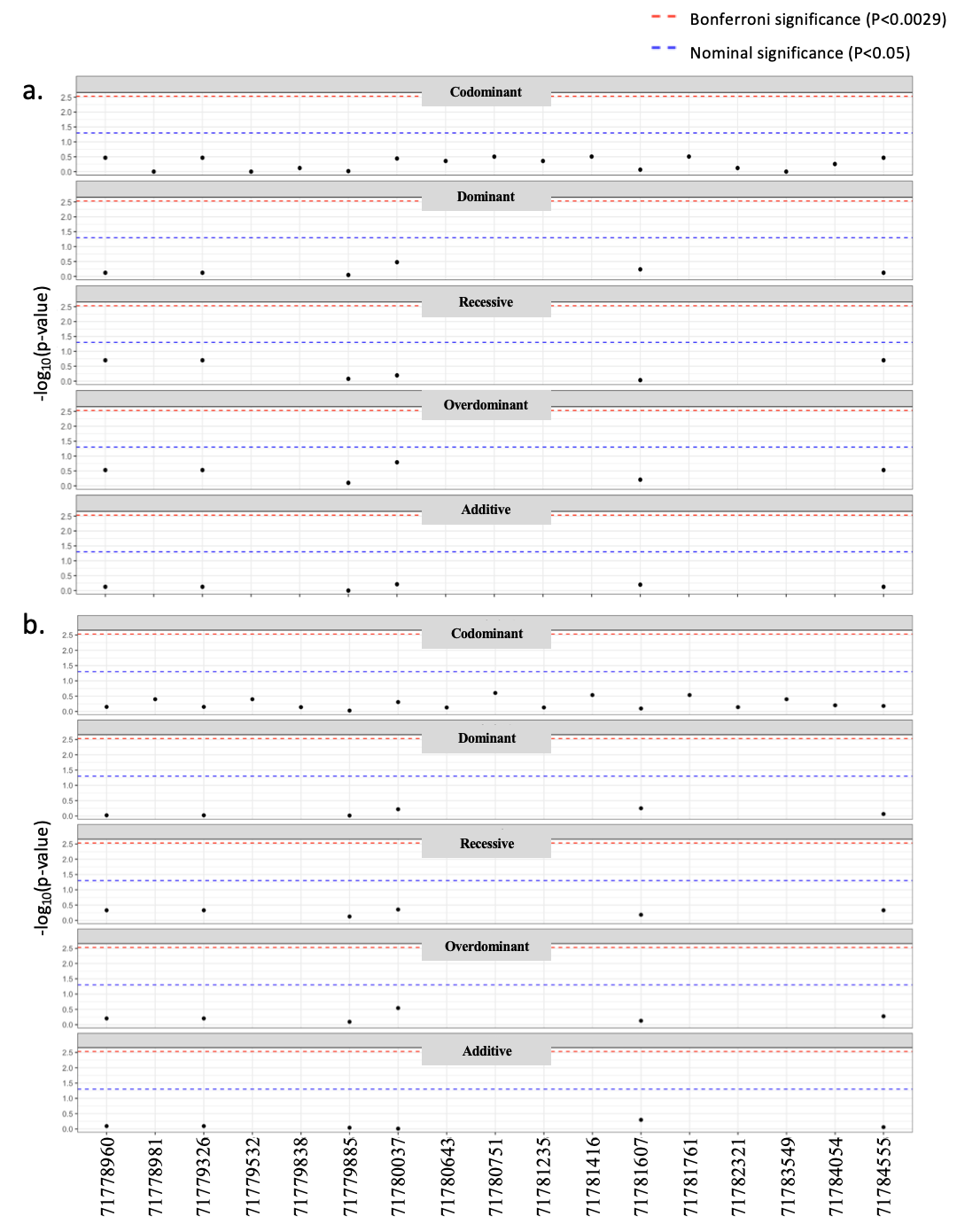
Supplementary 4: Chromosome analyses of significant SNPs on chromosome 15 before (a) and after (b) the addition of covariates (age, breed^†^, sex, and hydromorphone dosage rate) using generalizes linear model. ^†^ Iberian, Standardbred, & Arabian horses were excluded (n=4).

SNP location
